# Attenuation of Scopolamine-Induced Amnesia via Cholinergic Modulation in Mice by Synthetic Curcumin Analogs

**DOI:** 10.3390/molecules27082468

**Published:** 2022-04-11

**Authors:** Haya Hussain, Shujaat Ahmad, Syed Wadood Ali Shah, Abid Ullah, Niaz Ali, Mazen Almehmadi, Manzoor Ahmad, Atif Ali Khan Khalil, Syed Babar Jamal, Hanif Ahmad, Mustafa Halawi

**Affiliations:** 1 Department of Pharmacy, Shaheed Benazir Bhutto University Sheringal Dir (Upper), Dir 18000, Pakistan; haya@sbbu.edu.pk (H.H.); abid@sbbu.edu.pk (A.U.); 2 Department of Pharmacy, University of Malakand Dir (Lower), Chakdara 18800, Pakistan; 3 Department of Pharmacology, Institute of Basic Medical Sciences (IBMS), Khyber Medical University, Peshawar 25000, Pakistan; dr.niazali@kmu.edu.pk; 4 Department of Pharmacology, College of Medicine, Shaqra University, Shaqra 15526, Saudi Arabia; 5 Department of Clinical Laboratory Sciences, College of Applied Medical Sciences, Taif University, P.O. Box 11099, Taif 21944, Saudi Arabia; dr.mazen.ma@gmail.com; 6 Department of Chemistry, University of Malakand Dir (Lower), Chakdara 18800, Pakistan; manzoorahmad@uom.edu.pk (M.A.); akhund83@gmail.com (H.A.); 7 Department of Biological Sciences, National University of Medical Sciences, Rawalpindi 46000, Pakistan; atif.khalil7799@gmail.com (A.A.K.K.); babar.jamal@numspak.edu.pk (S.B.J.); 8 Department of Medical Laboratory technology, College of Applied Medical Sciences, Jazan University, Jazan 45142, Saudi Arabia; halawim@jazanu.edu.sa

**Keywords:** curcumin analogs, Alzheimer’s disease, amnesia, acetylcholinesterase, butyrylcholinesterase, docking, scopolamine, EPM, Y-maze, NORT, hippocampus

## Abstract

Alzheimer’s disease is an emerging health disorder associated with cognitive decline and memory loss. In this study, six curcumin analogs (**1a**–**1f**) were synthesized and screened for in vitro cholinesterase inhibitory potential. On the basis of promising results, they were further investigated for in vivo analysis using elevated plus maze (EPM), Y-maze, and novel object recognition (NOR) behavioral models. The binding mode of the synthesized compounds with the active sites of cholinesterases, and the involvement of the cholinergic system in brain hippocampus was determined. The synthesized curcumin analog **1d** (*p* < 0.001, *n* = 6), and **1c** (*p* < 0.01, *n* = 6) showed promising results by decreasing retention time in EPM, significantly increasing % SAP in Y-maze, while significantly (*p* < 0.001) enhancing the % discrimination index (DI) and the time exploring the novel objects in NORT mice behavioral models. A molecular docking study using MOE software was used for validation of the inhibition of cholinesterase(s). It has been indicated from the current research work that the synthesized curcumin analogs enhanced memory functions in mice models and could be used as valuable therapeutic molecules against neurodegenerative disorders. To determine their exact mechanism of action, further studies are suggested.

## 1. Introduction

Alzheimer’s disease (AD) is a neurodegenerative brain disease responsible for both mental and physical deterioration in patients, causing death. It is one of the common forms of dementia [1,2], characterized by serious short-term memory loss with cognitive decline, impaired reasoning and judgment, and communication difficulties followed by neurodegeneration, and it is considered a growing health issue worldwide [3]. It has been surveyed that 60–70% cases of AD were diagnosed with dementia [4], and about 46.85 million affected individuals with AD around the globe and it has been predicted that this figure will be two-fold by 2030, according to a report published in 2015 on Alzheimer’s disease [5]. The exact cause of AD is still a mystery to researchers; however, cholinergic deficiency has been considered one of the leading causes associated with cognitive decline and is correlated to AD [2].

Acetylcholine is a neurotransmitter responsible for the regulation of cognitive functions, and the centrally acting cholinergic system, maintains their level, which plays a vital role in the memory and learning process [2,6]. Acetylcholine is degraded by cholinesterases, which causes serious cholinergic deficiency leading to the onset of AD, and inhibitions of these enzymes remains a significant therapeutic target for AD [7]. The immediate response of altering enzyme activity remains the prime target for drug design; even with the increased usage of drugs, 47% of all current drugs inhibit the enzyme targets [8]. Based on cholinergic hypothesis, AChE inhibitors are the most prescribed drugs against the disease, as patients with AD have an apparent deficit in acetylcholine (ACh) and possess multifactorial and complex pathophysiology. Therefore, acetylcholinesterase inhibition is suggested to be a promising strategy [9].

**Figure 1:**
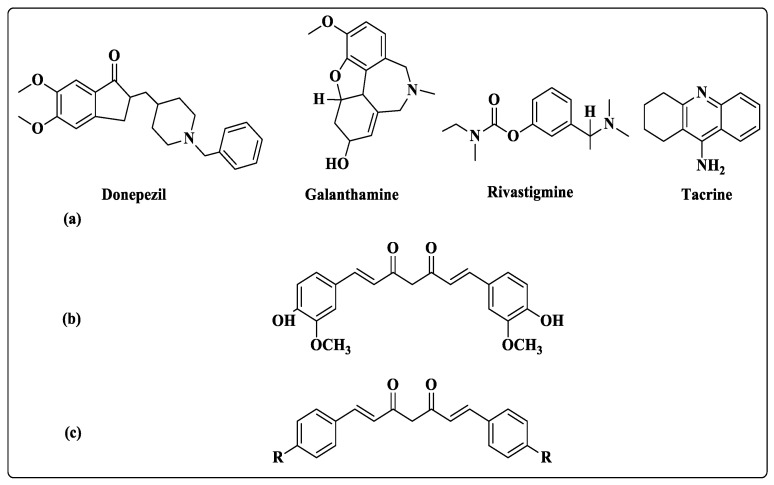
(**a**) Cholinesterase inhibitors (**b**) Chemical structure of natural curcumin (**c**) General structure of curcumin analogs.

Recent studies indicated the role of basal forebrain cholinergic systems in memory and cognition [10]. The cholinergic system plays a vital role in the memory retention process by maintaining the acetylcholine level [11]. The decreased level of acetylcholine causes memory impairment, which can be overcome with the management of cholinesterase inhibitors [12]. The activation of the cholinergic system by the administration of acetylcholinesterase inhibitors offers symptomatic aid in dementia. Currently, acetylcholinesterase inhibitors, donepezil, galantamine, rivastigmine and tacrine have been approved for symptomatic therapy of AD, which were reported recently [13,14,15] (Figure 1a). The available cholinesterase inhibitors only manage to prevent the progress of the disease but are unable to cure it because they lose long-term efficacy. They also cause severe side effects and it is why there is an urgent need for designing effective anti-AD molecules.

Curcumin is one of the most important constituents of the curcuminoid family and is naturally found in *Curcuma longa* L. [16] (Figure 1a). Curcumin showed a wide range of biological applications and has been used for centuries as a dietary pigment and as a spice [17]. Recently, it was reported that curcumin and curcuminoids have memory-enhancing effects in rats [18]; more importantly, they played a crucial role as a remedy for Alzheimer’s disease and other neurodegenerative diseases [2,19,20]. It has been reported that β-diketone moiety is responsible for many pharmacological activities and plays a vital role in the reactivity of curcumin analogs [21]. Curcumin analogs with active methylene group (-CH2-) between two carbonyl groups exhibited significant potential against AD [3]. The aim of this research was to design symmetric synthetic curcumin analogs with di-carbonyl moiety, encouraging the cascade of our previously published work on mono-carbonyl curcumin analogs [2], and evaluate them in behavioral mice models for memory-enhancing effects as possible alternative therapeutics molecules for neurodegenerative diseases like AD.

## 2. Results

**Scheme 1:**
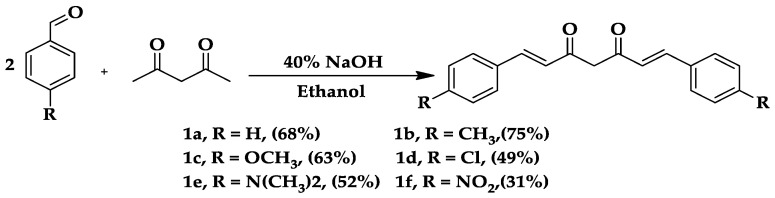
Synthesis of curcumin analogs **1a**–**1f**.

In this research study, six symmetrical synthetic curcumin analogs (**1a**–**1f**) with substituted functional groups on aromatic rings were synthesized from acetyl acetone treated with substituted aldehydes at room temperature in the presence of ethanol as solvent (Scheme 1). The compound **1a** with no substitution on the aryl ring, **1b** with methyl substituent, **1c** with methoxy substituent, and **1e** with N,N’-dimethyl amino substituent were synthesized in good yields. These substituents have an electron-donating effect and increased the reactivity of the compounds, while **1d** and **1f** have deactivating substituents that decreased the product yield.

### 2.1. In Vitro Cholinesterase Activity

The in vitro anti-cholinesterase activity of the synthesized curcumin analogs was determined against cholinesterases (AChE and BuChE), as shown in Table 1. Compounds **1d**, **1c**, and **1b** showed comparable results and higher AChE inhibition with IC_50_ values of 112.52 nM, 467.18 nM and 733.84 nM, respectively, while compound **1a** with IC_50_ 2615.42 nM, **1e** with IC_50_ 3267.95 nM, and **1f** with IC_50_ 5839.96 nM showed mild enzyme inhibition activities compared to standard donepezil. Donepezil was used as the standard with IC_50_ of 9.31 nM. Similarly, against the BuChE enzyme, the synthesized curcumin analogs were evaluated in a similar way as for acetylcholinesterase, and it was revealed that the tested samples showed lower enzyme inhibition activities against BuChE than they did against the AChE enzyme. Selectivity for AChE with IC_50_ values calculated for **1d** were 378.43 nM, followed by **1c** with IC_50_ 1356.14 nM, and **1b** with IC_50_ 2159.08 nM, while compound **1a** with IC_50_ 5347.16 nM, **1e** with IC_50_ 6635.82 nM, and **1f** with IC_50_ 9664.71 nM have weak butyrylcholinesterase inhibition activities compared to standard donepezil. The IC_50_ value obtained for donepezil remained 33.65 nM.

### 2.2. Molecular Docking

MOE-Dock protocol was used for the prediction of an interaction between ligX application, ligand molecules, and AChE and BuChE within the MOE package [22]. Both the cholinesterases were docked with (**1a**–**1f**) compounds via the MOE tool. All the synthesized compounds showed good binding affinity with target proteins. The IC_50_ values were parallel to the binding modes of compounds.

#### Validation of Docking for Anticholinesterases

**Figure 2:**
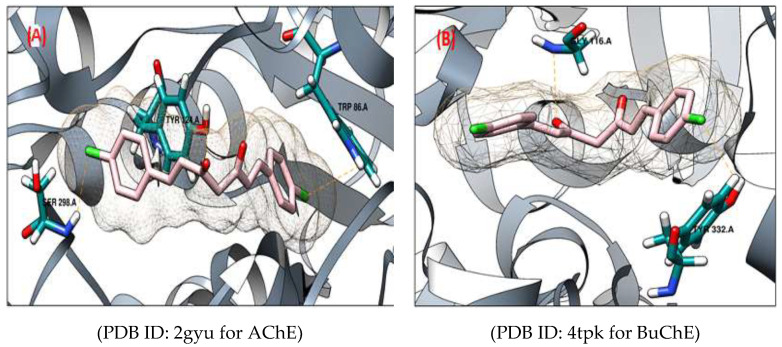
3D binding mode of compound (**1d**) against cholinesterase (**A**) AChE and (**B**) BuChE protein.

Synthesized curcumin analogs, as co-crystallized ligands, were re-docked in the cholinesterase inhibitor (PDB ID: 2gyu) binding cavity after removal from the active sites. The incorporated docking protocol for the tested compounds, after the observed RMSD 1.618 Å value, was validated and MOE-Dock was determined to be a reliable method. The compound **1d** exhibited a promising docking score (−12.089) for AChE, and both the active site residues Trp86 and Ser298 showed interaction with the chlorobenzene moieties, while the third active site residue Tyr124 made a hydrogen bond with the di-carbonyl moiety (Figure 2A) Table 2. Similarly, **1d** was also active against BuChE, interacting with the residues of two active sites with a docking score of −10.962, in which Tyr332 interacted with chlorobenzene moiety, while Gly116 interacted with the di-carbonyl moiety via hydrogen bonding (Figure 2B) and Table 2.

The data for binding energy values and docking scores for docked targets with the compounds (**1a**–**1f**) are shown in Table 2. The data interpretation of binding interactions of synthesized compounds with docked targets showed that the tested compounds were accurately docked into the active site residues and showed significant interactions against both cholinesterases (AChE and BuChE).

### 2.3. In Vivo Behavioral Study

The synthesized curcumin analogs were evaluated using EPM, Y-maze and NORT behavioral models for investigation of the memory-enhancing potential.

#### 2.3.1. Elevated Plus Maze

**Figure 3:**
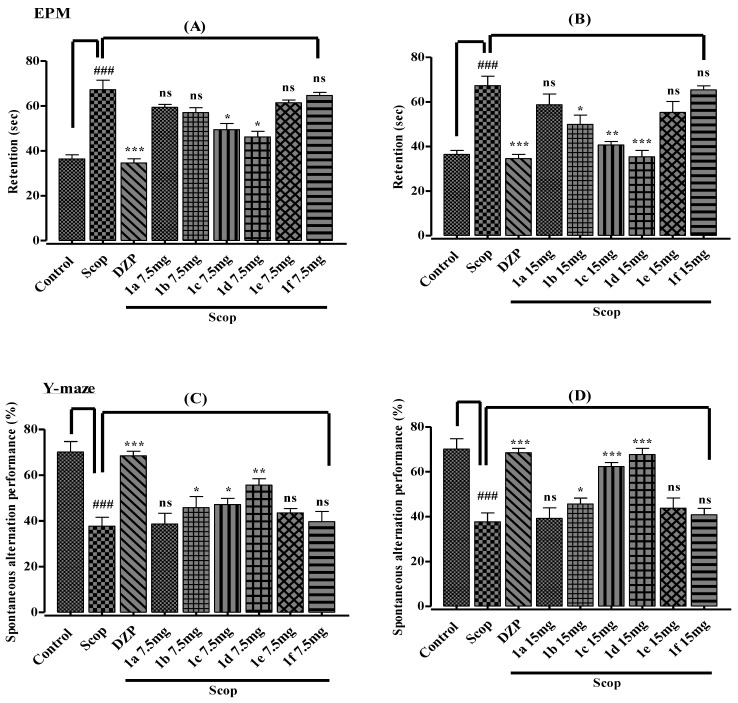
Effects of synthesized curcumin analogs (**1a**–**1f**), (**A**) Transfer latency (seconds) at 7.5 mg/kg of the tested compounds and (**B**) Transfer latency (seconds) at 15 mg/kg of the tested compounds in EPM behavioral mice model versus scopolamine treated groups (1 mg/kg); (**C**) %Spontaneous alternation performance at 7.5 mg/kg of the tested compounds and (**D**) %Spontaneous alternation performance at 15 mg/kg of the tested compounds versus scopolamine (1 mg/kg) treated group in Y-maze behavioral mice model. Data were presented in (mean ± SEM), *n* = 6. ### *p* < 0.001 vs. normal control. Significantly different values were compared with amnesic group; ***, **, * and ns represents *p*-value < 0.001, <0.01, <0.05, >0.05 respectively.

In the elevated plus maze (EPM) paradigm, synthesized curcumin analogs showed promising results (Figure 3A,B). There was a marked amnesia induced in mice after 30 min of the last dose on day 7 upon administration of scopolamine 1 mg/kg i.p, and a significantly higher transfer latency time (TLT) in seconds was recorded in comparison to the vehicle treated group. Pretreatment with donepezil significantly altered and decreased TLT (34.57 ± 1.93 s) (*p* < 0.001) when compared to the amnesic group.

Similarly, pretreatment with synthesized compounds in both doses for 7 days, in which compound **1d** had significantly reduced TLT in seconds (*p* < 0.05) at 7.5 mg/kg and (*p* < 0.001) at 15 mg/kg, reversed the amnesia. For the other compounds: **1c** with reduced TLT in seconds (*p* < 0.05) at 7.5 mg/kg and (*p* < 0.01) at 15 mg/kg, respectively, showed a weak response, while **1a** (*p* > 0.05) was non-significant, **1b** (*p* > 0.05) was non-significant at 7.5 mg/kg and (*p* < 0.05) showed poor response at 15 mg/kg. Similarly, **1e** and **1f** were non-significant at both doses, respectively.

#### 2.3.2. Y-Maze Test

The synthesized curcumin analogs were evaluated for anti-amnesic effect using the Y-maze mice model, which showed significantly higher memory-enhancing potential, Figure 3 (C and D). Scopolamine administration caused serious amnesia by decreasing the percent spontaneous alternation performance to 37.66 ± 3.94 (*p* < 0.001) in comparison with normal controls (70.16 ± 4.58). The standard drug donepezil with a % SAP of 68.50 ± 1.95 (*p* < 0.001) and the treated compounds **1d** at both doses have significantly increased the % SAP at 55.66 ± 2.82 (*p* < 0.01) and 67.66 ± 2.84 (*p* < 0.001), respectively, when compared to the scopolamine treated amnesic group. The memory-enhancing effects were observed to be comparable for compound **1c** with a % SAP of 47.16 ± 2.77 (*p* < 0.05) at 7.5 mg/kg, and was enhanced significantly at 15 mg/kg with a % SAP of 62.33 ± 184 (*p* < 0.001), while **1a**, **1b** showed poor response (*p* < 0.05), and **1e**, **1f** (*p* > 0.05) were non-significant at both doses, respectively. The curcumin analogs **1d** and **1c** have showed their effectiveness against memory impairment in amnesic mice.

#### 2.3.3. Novel Object Recognition Test

**Figure 4:**
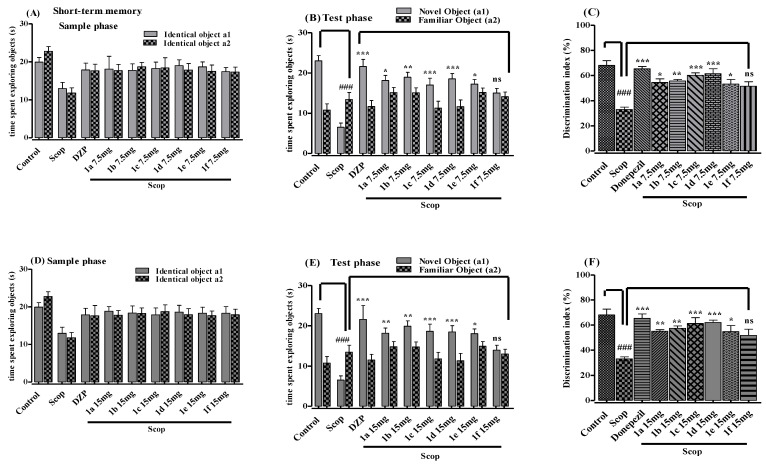

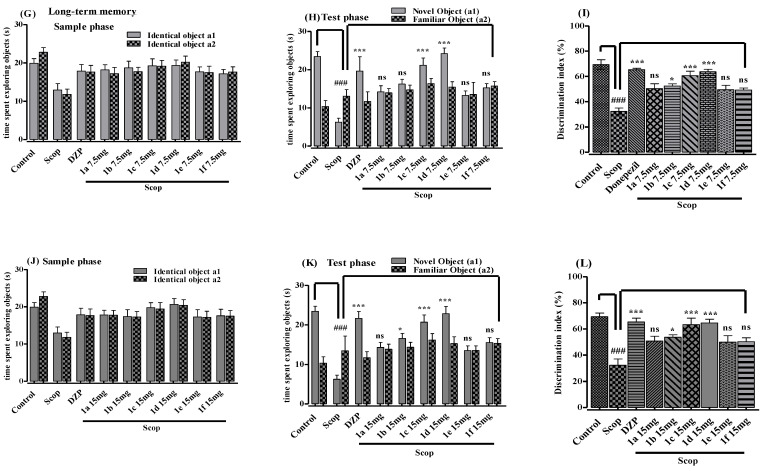
The effect of synthesized curcumin analogs for the evaluation of memory (**1a–1f**) in NORT (**A**) Time spent in the sample phase, (**B**) Time spent in the test phase, (**C**) Discrimination index (%) were measured in treated groups (7.5 mg/kg) versus scopolamine (1 mg/kg) treated group. (**D**) Time spent in the sample phase, (**E**) Time spent in the test phase, (**F**) Discrimination index (%) were measured in treated groups (15 mg/kg) versus scopolamine (1 mg/kg) treated group for measuring short-term memory in NORT behavioral mice model; (**G**) Time spent in the sample phase, (**H**) Time spent in the test phase, (**I**) Discrimination index (%) were measured in treated groups (7.5 mg/kg) versus scopolamine (1 mg/kg) treated group. (**J**) Time spent in the sample phase, (**K**) Time spent in the test phase, (**L**) Discrimination index (%) were measured in treated groups (15 mg/kg) versus scopolamine (1 mg/kg) treated group for measuring long-term memory in NORT behavioral mice model. Results were presented in (mean ± SEM), *n* = 6. ### *p* < 0.001 vs. normal control. Significantly different values were compared with amnesic group; ***, **, * and ‘ns’ represents *p*-value < 0.001, <0.01, <0.05, >0.05 respectively.

The synthesized curcumin analogs (**1a**–**1f**) for memory-enhancing potential using the NORT mice model were evaluated, and the results are presented in Figure 4. In the short-term memory, there was no significant change observed in sample phase in the exploration time for both identical objects when treated with all sample compounds. There was a significantly higher exploration time for the novel object than the identical object recorded in the test phase when treated with synthesized compounds at 7.5 and 15 mg/kg and standard donepezil at 2 mg/kg. Pretreatment with donepezil has significantly enhanced the time spent in seconds (21.59 ± 1.44 (*p* < 0.001)) exploring the novel object with 65.26% DI and it was reduced for the familiar object (11.49 ± 1.39 s 32.32% DI) as compared to the amnesic group. Synthesized curcumin analogs **1c** with a discrimination index of 60.16% (*p* < 0.001) and 61.25% (*p* < 0.001) at both doses, respectively, and **1d** with 61.43% (*p* < 0.001) and 62.03% (*p* < 0.001) showed promising results with a significantly higher exploration time compared to the amnesic group. Similarly, in this study, compound **1a** with a DI of 54.53% (*p* < 0.05) and 55.10% (*p* < 0.01) at both doses, respectively, showed promising results, and **1b** with a DI of 55.68% (*p* < 0.01) and 57.47% (*p* < 0.01) showed significant results at both doses, respectively, while **1e** and **1f** showed no promising potential in comparison with the amnesic group. After 24 h of the short-term memory evaluation, in terms of long-term memory, the synthesized curcumin analogs were tested and the results indicated that **1c** and **1d** showed a significant improvement in comparison with the amnesic group, with a DI of 60.58% (*p* < 0.001), 62.28% (*p* < 0.001), and 63.78% (*p* < 0.001), 64.61% (*p* < 0.001) at both doses, respectively. Compound **1b** demonstrated moderate memory-enhancing activity with a DI of 52.45% and 53.61% (*p* < 0.05) at both doses, respectively, while **1a**, **1e** and **1f** showed no significant change.

### 2.4. Biomarker Study

The synthesized compounds after in vitro and in vivo study were subjected into an ex vivo biomarker study to check the activity of AChE and BuChE in the hippocampi of mice brains.

#### Effect of Synthesized Curcumin Analogs on AChE and BuChE Activity in the Brain Hippocampus

**Figure 5:**
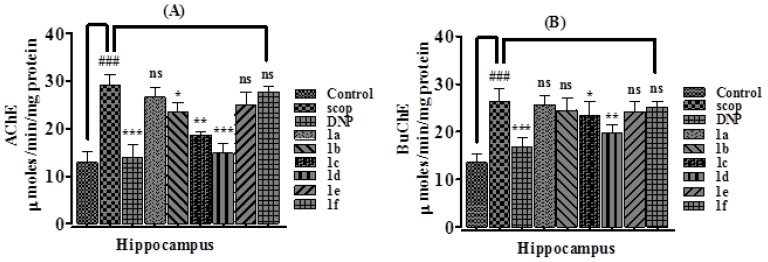
Ex vivo anticholinesterase effect of synthesized curcumin analogs (**1a–1f**) in AChE (**A**), BChE (**B**) in the hippocampus region of mice brains versus scopolamine treated group. Data were presented in (mean ± SEM), *n* = 6. ### *p* < 0.001 vs. normal control. Significantly different values were compared with amnesic group; ***, **, * and ns represents *p*-value < 0.001, < 0.01, <0.05, > 0.05 respectively.

In this study, the cholinesterases’ (AChE and BuChE) inhibitory activity was investigated in the hippocampi of mice brains. Scopolamine administration notably (*p* < 0.001, *n* = 6), increased AChE activity compared to the control group. The increased AChE activity caused the degradation of acetylcholine and produced cholinergic deficiency, which results in amnesia. The compound **1d** (*p* < 0.001, *n* = 6), **1c** (*p* < 0.01, *n* = 6), **1b** (*p* < 0.05, *n* = 6) and standard donepezil (*p* < 0.001, *n* = 6) markedly reduced the increased activity of AChE induced by scopolamine (Figure 5A). However, among these groups, only **1d** (*p* < 0.01, *n* = 6) and **1c** (*p* < 0.05, *n* = 6) decreased the increased BuChE activity induced by scopolamine, showing their selectivity towards AChE (Figure 5B).

## 3. Discussion

Curcumin exhibits low toxicity and good bioactivities [23] and it has been reported that curcumin and curcumin analogs have the potential to inhibit the toxicity induced by nicotine in the lungs [24]. The synthesized curcumin analogs showed a safe profile in the acute toxicity study.

The cholinesterase inhibition by the synthesized compounds were consistent with the reported cholinesterase inhibitory activity of curcumin [20]. The current research study indicated the higher cholinesterase (AChE and BuChE) inhibitory potentials of curcumin analogs, specifically **1d** and **1c**. The inhibition of the cholinesterases by the synthesized curcumin analogs was supported by the molecular docking study, according to MOE-Dock protocol. The cholinesterase activity against both cholinesterases (AChE and BuChE) were predicted by interacting ligand molecules, ligX application and cholinesterases [22].

The docked pose of both targets, AChE and BuChE, indicated that compound **1d** has affinity with the target proteins, making strong interactions with chlorobenzene and di-carbonyl moiety, which predicted significant cholinesterase inhibition. The docking of the synthesized compounds were successfully correlated with the three dimensional crystallographic structures of the compound, specifically **1d,** as observed against AChE and BuChE, given the in vitro results of **1d** structure similarities with the calculated binding affinities of (−12.089 kcal/mol) and (−10.962 kcal/mol), respectively [25]. The compound 1d with chloride substituent on a phenyl ring showed higher enzyme inhibitory potential when compared to other analogs, which was parallel with the previous finding [26].

The binding capability of the synthesized curcumin analogs and their enzyme inhibitory potentials were consistent with the binding interactions between amino acids and different functional groups of curcumin and their analogs [27,28]. This study suggested that these compounds could be therapeutic candidates against neurodegenerative diseases.

Elevated plus maze is considered a reliable behavioral mice model for evaluation of memory in mice [29]. The acquisition and retention of memory in spatial long-term memory has been determined by an EPM behavioral learning task [30]. The improvement in the memory of mice by the synthesized compounds revealed from the EPM mice paradigm with the reduction of transfer latency in the retention session was significantly different from the acquisition session. The pretreatment of synthesized curcumin analogs have significantly attenuated the memory deficits induced by scopolamine administration in mice [31]. The potential benefits of curcumin analogs were revealed from the memory-enhancing effects in the elevated plus maze (EPM) mice model study. The synthesized curcumin analogs significantly reversed the memory deficits caused by scopolamine, and protected neuronal degeneration and enhanced memory, which is parallel with the reported studies [20,32].

Y-maze is a behavioral mice model based on sequential two choice discriminations that inherently motivates animals to explore unknown environments [33]. Y-maze has a simple structure and convenient operation, due to which more animal experiments have adopted the Y-maze model for exploring objects and measuring memory in mice [34]. The Y-maze paradigm presented percent spontaneous alternation behavior in mice as described in the reported studies [35,36]. The compound **1c** and **1d** significantly increased the % SAP, which is consistent with the reported studies [5,37].

The recognition of previously explored stimulus in the behavioral mice models form the focus of research on human amnesia [38]. The novel object recognition test is a common behavioral model evaluating learning and cognition aspects of animal behavior. It can be completed in just a few days and is a simple technique with habituation, familiarization and test sessions. In the familiarization phase, two identical objects were explored by mice; in the test phase, on object is replaced by a novel object [39]. The time spent exploring the novel object was significantly higher when the mice were t treated with synthesized compounds and donepezil in the test phase and showed a high discrimination index. The long-term memory in the mice was also retained when treated with donepezil and synthesized curcumin analogs, indicating an improvement in learning and memory. These results from the behavioral assays were consistent with the in vitro cholinesterase inhibition activity which was supported by the molecular docking approach and consistent with the previous research [5,40]. 

The cholinergic depletion caused memory impairment, as scopolamine did in the amnesic group, by increasing the activity of cholinesterases (AChE and BuChE), which degraded acetylcholine in the synaptic cleft. The synthesized curcumin analogs and donepezil retained memory by significantly reducing the cholinesterases activity in the brain hippocampus, as revealed in the behavioral assays. The inhibitors of cholinesterases enhanced the acetylcholine level by inhibiting AChE, which increased both the transmission of nerve conduction and the period of transmission [41]. In this tudy, the increased level of acetylcholine and the decreased activity of AChE in the hippocampi of mice brains following treatment with synthesized curcumin analogs, showed the potential role of synthesized curcumin analogs as cholinesterase inhibitors [18,20]. 

## 4. Materials and Methods

Chemicals and solvents were from Merck, Sigma Aldrich, Darmstadt, Germany, which were of analytical grade and obtained from local markets. Different solvent systems were used for checking the purity of the compounds, giving single spot in ethyl acetate and n-hexane. Thin-layer chromatography (TLC) plates (Merck 60F_254_) on silica gel were used for monitoring the progress of reaction. ^1^H-NMR spectra (see Supplementary) were obtained using FT Spectrometer (Bruker Varian Mercury 300 MHz, Billerica, MA, USA) in CDCl_3_. Mass spectra (MS) were determined using water: Micromass ZMD connected to Varian MAT 312 double focusing mass spectrometer connected to DEC-PDP 11/34 computer system (Milford, CT, USA). The synthesized compounds were docked using Molecular Operating Environment (MOE) software package (http://www.chemcomp.com/) (access date: 5 April 2021).

### 4.1. Methods

#### 4.1.1. General Procedure for the Synthesis of Curcumin Analogs (**1a**–**1f**)

A series of symmetrical curcumin analogs were synthesized by treating substituted aldehydes with acetyl acetone. Substituted aldehydes (2 mmol) were treated with acetyl acetone (1 mmol) in 250 mL reaction flask using ethanol (15 mL) as solvent, and NaOH 40% solution (10 mL) was added. The reaction mixture was stirred continuously for 2 h and the reaction progress was monitored by TLC. At the end, HCl (1:1) aqueous solution was added to neutralize the catalyst, and finally, the filtered dried precipitate of the reaction was recrystallized in ethanol or ethyl acetate [42].

##### Synthesis of (1E,6E)-1,7-diphenylhepta-1,6-diene-3,5-dione (**1a**)

Yield: 68%, yellow crystalline powder, solubility: chloroform, ethyl acetate, melting point: 151–154 °C. Rf value: 0.70 in ethyl acetate hexane eluents (3:7). ^1^H-NMR (CDCl_3_, 300 MHz): δ 7.8 (d, 2H) 7.6 (d, *J* = 15.9 Hz, 2H, Ar), 7.28 (d, *J* = 6.6 Hz, 4 H Ar), 7.09 (d, 2H,). 7.64 (d, *J* = 15.8 Hz, 4H Ar), 6.9 (d 2H) 4.0 (s, 2H). HR-MS *m/z*: 277.1184 (M + 1)^+^, calcd for C_19_H_16_O_2_ 276.1150. ^13^CNMR, and IR was reported by [43].

##### Synthesis of (1E,6E)-1,7-di-p-tolylhepta-1,6-diene-3,5-dione (**1b**)

Yield: 75%, yellow crystals, melting point: 179–183 °C, solubility: chloroform, ethyl acetate, Rf value: 0.76 in ethyl acetate hexane eluents (3:7). ^1^H-NMR (CDCl_3_, 300 MHz): δ 7.76 (s, *J* = 15.9 Hz, 2H), 7.037 (d, 2H) 7.54 (m, 4H Ar), 7.24 (m, 4H Ar), 7.06 (d, *J* = 15.9 Hz, 2H), 3.87 (s, 2H), 2.41 (s, 6H). IR ν_max_ (cm^−1^): 3340; 2927;1880; 1717; 1650; 1593; 1504; 1437; 1403; 1339; 1294; 1276; 1170; 1075; 990; 977. HR-MS *m/z*: 305.1497 (M + 1)^+^, calcd for C_21_H_20_O_2_ 304.1463.

##### Synthesis of (1E,6E)-1,7-bis(4-methoxyphenyl)hepta-1,6-diene-3,5-dione (**1c**)

Yield: 63%, yellow crystals, melting point: 164–167 °C, solubility: chloroform, ethyl acetate, Rf value: 0.44 in ethyl acetate hexane eluents (3:7). ^1^H-NMR (CDCl_3_, 300 MHz): δ7.5 (d, *J* = 15.8 Hz, 2H), 7.6 (d, *J* = 8.8 Hz, 2H), 6.9 (d, *J* = 8.8 Hz, 2H), 4.91 (s, 2H), (s, 1H), 3.87 (s, 6H). ^13^CNMR and IR was reported by [43].

##### Synthesis of (1E,6E)-1,7-bis(4-chlorophenyl)hepta-1,6-diene-3,5-dione (**1d**)

Yield: 49%, yellow crystals, melting point: 166–168 °C, solubility: chloroform, ethyl acetate, Rf value: 0.72 in ethyl acetate hexane eluents (3:7). ^1^H NMR (CDCl_3_, 300 MHz): δ7.72 (d, *J* = 15.8 Hz, 2H), 7.67 (d, *J* = 8.4 Hz, 4H), 7.2 (d, *J* = 8.4 Hz, 4H), 4.56 (s, 2H). HR-MS *m/z*: 346.0341 (M + 1)^+^, calcd for C_19_H_14_Cl_2_O_2_ 344.0371. ^13^CNMR, and IR data was reported by [43].

##### Synthesis of (1E,6E)-1,7-bis(4-(dimethylamino)phenyl)hepta-1,6-diene-3,5-dione (**1e**)

Yield: 52%, reddish powder, melting point: 194–198 °C, solubility: chloroform, ethyl acetate, Rf value: 0.51 in ethyl acetate hexane eluents (3:7). ^1^H-NMR (CDCl_3_, 300 MHz): δ 7.77 (d, 2H), 7.74(d, 4H, Ar), 7.32(d, 4H, Ar), 6.59 (d,2H), 6.53 (s, 2H), 3.01 (s, 6H) 3.09(s, 3H). HR-MS *m/z*: 363.2028 (M + 1)^+^, calcd for C_23_H_26_N_2_O_2_ 362.1994. ^13^CNMR and IR data was reported by [44].

##### Synthesis of (1E,6E)-1,7-bis(4-nitrophenyl)hepta-1,6-diene-3,5-dione (**1f**)

Yield: 31%, brown powder, melting point: 108–113 °C, solubility: ethyl acetate, Rf value: 0.11 in ethyl acetate hexane eluents (3:7). ^1^H-NMR (CDCl_3_, 300 MHz): δ 8.43 (d, *J* = 15.8 Hz, 4H), 8.40 (d, *J* = 8.4 Hz, 4H), 7.74 (d,2H) 7.28 (d, *J* = 8.4 Hz, 2H), 4.58 (s, 2H). IR ν_max_ (cm^−1^): 3359; 2931; 1727; 1660; 1650; 1597; 1508; 1431; 1407; 1342; 1298; 1272; 1165; 1070; 995; 971.

### 4.2. In Vitro Cholinesterase Assay

The in vitro cholinesterase assay of the synthesized curcumin analogs were carried out according to the Ellman’s method with slight modification [45]. The enzymes AChE and BuChE were obtained from electric eel and equine serum, respectively. Test samples were dissolved in methanol then diluted in phosphate buffer (0.1 M) from 1000 to 62.5 µg/mL concentrations. The dilution of 518 U/mg AChE and 7–16 U/mg BuChE was accomplished in 0.1 M (pH 8.0) phosphate buffer. The final concentrations of AChE 0.03 U/mL and 0.01 U/mL of BuChE were achieved. The substrate of 0.5 mM acetylcholine iodide, 0.2273 mM DTNB and 0.5 mM butyrylcholine iodide solution was made and maintained separately at 8 °C in vials. The experiment was started by adding 5 µL enzyme solutions to cuvette, then 205 µL test sample and 5 µL DTNB reagents were added. Substrate solution (5 µL) was added, and the solution mixture was kept in a water bath for 15 min at 30 °C. Spectrophotometric absorbance was recorded at 412 nm at 30 °C with a reaction time of 4 min. The donepezil was kept as positive control and the experiment was carried out in triplicate. Percent enzyme inhibition was calculated using formula:

$$V=\frac{∆Abs}{∆t}$$

where V = rate of change of absorbance; % enzyme inhibition = 100—% enzyme activity; % enzyme activity = 100 × V/V_max_, V_max_ = enzyme activity.

### 4.3. Molecular Docking Study

The protein data bank (PDB IDs: 2gyu and 4tpk) was used as the source for the 3-D structures of AChE and BuChE in this study, and the structures were refined using 3D protonation by removing water molecules of the protein molecules. The MOE software package (http://www.chemcomp.com/), access date: 5 April 2021, was applied for energy minimization of protonated (3D) structures. The current geometry in chiral constraint of MMFF94X + Solvation (force field) 0.05 gradients were set as the parameters for energy minimization. The energy minimization was terminated by default when the root-mean-square gradient fell below 0.5 [46]. The minimized protein structures (final product) were subjected to simulation of molecular docking.

Ligands were recaptured utilizing MOE Builder application [47] and just like the protein, the MOE apparatus was run for energy minimization of the structures and were spared in .mol2 arranged and traded in a single database of (.mdb) records. At that point, MOE-Dock was at long last utilized for molecular docking of the arranged database of ligand structures [48].

#### 4.3.1. *Receptors Preparation*

The donepezil inhibitor made from a complex of homodimer (PDB ID: 4EY7) with AChE of 2.35 Å resolutions and tacrine inhibitor of 2.10 Å resolutions, (PDB ID: 4BDS) and a monomer complex with human BuChE was used. Human AChE chain B was chosen for docking implementation and the missing residues were adjusted under MOE. The interacted water molecules were retained while others were deleted. The energy minimization was attained after adding hydrogen atoms in the complex structures [45].

#### 4.3.2. *Re-Docking Setup*

The MOE software was found validated after re-docking. The fitness of each re-docked pose in the active sites of AChE and BuChE was determined after incorporating the co-crystalized ligand on the basis of root-mean-square deviation [45].

### 4.4. Acute Toxicity Study

The synthesized compounds were tested for acute toxicity to get a safe dose for in vivo behavioral studies. The samples (**1a**–**1f**) were administered orally to different groups of animals in two stages. Animals were treated with 1.875, 3.75, 7.5, 15, 30, 50, 75 mg/kg doses in stage-I, and in stage-II, treated with 5, 15, 30, 50, 75, 100, 150 mg/kg doses of the synthesized compounds. Immediately after dosing, the mice were observed for 24 h for toxicity symptoms, such as tremors, lacrimation, salivation, convulsions, sedation, motor activity, loss of righting reflex, hypnosis, and diarrhea and muscle spasm. The mice were observed for 72 h for mortality. All the samples were safe and non-toxic up to 75 mg/kg in stage-I and 150 mg/kg doses in stage-II. Therefore, a dose of 7.5 mg/kg, which was 1/10th of 75 mg/kg, and 15 mg/kg dose, which was 1/10th of 150 mg/kg treated dose, according to OECD guidelines, were selected as the appropriate doses for the behavioral activities [5].

### 4.5. In Vivo Analysis

The synthesized compounds were evaluated for possible memory-enhancing potentials after the preliminary in vitro cholinesterase inhibition assays, according to the standard procedures.

#### 4.5.1. Animals and Dosing

This study was conducted according to approval from the “Departmental Ethical Committee (SBBU/IEC-20–02)” in compliance with Scientific Procedure Issue-I of the University of Malakand 2008 animal Bye-Laws. A total of sixteen groups of mice, with six in each group, were used in the experiment. Vehicle treated group (Normal control group) were administered 10 mL/kg (p.o) of normal saline. The scopolamine treated (amnesic) group were administered 1 mg/kg (i.p) of scopolamine. Donepezil treated were administered 2 mg/kg (p.o) of donepezil. Sample treatment groups were administered 7.5 mg/kg and 15 mg/kg (p.o) of synthesized compounds, respectively. All groups were administered with various doses continually for 7 days, and on day 7, 60 min after the last dose of donepezil or tested samples, 1 mg/kg (i.p) dose of scopolamine was administered to each animal, except the vehicle treated group, and 30 min after the scopolamine dose, the cognitive paradigms were evaluated [49].

#### 4.5.2. Elevated Plus Maze

Elevated plus maze paradigm is an important behavioral model for evaluation of anti-amnesic potentials in mice [50]. This apparatus comprised of two open and two closed arms with dimensions of 16 cm × 5 cm × 12 cm and elevated 25 cm from the floor on a wooden stand. The EPM was designed in plus shape from two acrylic sheets having a central platform with dimensions of 5 cm × 5 cm. Initially, mice were placed in one of the open arms in such a way that it was directed away from the central platform. Then TLT was recorded after the mice moved to the closed arm, with their four legs from the open arm and then the mice were returned to their home cage. The time of exploration of the apparatus for each animal was 90 s. The mice, if they failed to find the closed arm in a specific time, were then gently moved to the closed arm, and a time of 90 s was assigned for that specific animal as tail and latency time. The TLT was noted 45 min after scopolamine administration. The mice explored the apparatus and returned to their home cage, and after 24 h of scopolamine administration, TLT was noted again. Reduction of TLT indicated memory-enhancing effect of the test samples. The inflexion ratio (IR) was calculated as:

$$\mathrm{IR}=\frac{L0-L1}{L0}$$

where L1 presents the initial transfer latency (s), and L0 is the retention transfer latency (s).

#### 4.5.3. Y-Maze Test

The synthesized compounds were evaluated for anti-amnesic activity using Y-maze behavioral mice model, according to standard procedure [5]. This apparatus is designed in a Y-shape with equal three arms, and these arms were expressed by A, B, and C for convenience. The dimensions of the arms were 15.5 cm high, 6 cm wide, 20 cm long and connected with each other with an angle of 120°. This test was conducted for a 5 min duration for each animal. The order and number of arm entries made by each animal after placing in one arm were recorded. Complete arm entry in any given arm was considered to be when the hind paws were completely inside, and the alternation was the consecutive arm entries into three different arms by each mouse. Ethanol 70% *v*/*v* solution was used to clean the Y-maze arena to avoid olfactory cues between each test. At the end of day 7, escape latency (seconds) for each animal was recorded. Mice were freely allowed to explore the objects when they were initially placed at the center of the apparatus. The formula was used to calculate the %SAP by recording the same arm returns (SAR), alternate arm returns (AAR) and the number of arm entries.

$$SAP\;\left(\%\right)=\frac{total\;alterations}{total\;arm\;entries}—2\;\times \;100$$

#### 4.5.4. Novel Object Recognition Test (NORT)

In this study, the memory-enhancing potentials of synthesized compounds were determined using novel object recognition test mice model. Dimensions of the apparatus were 40 cm × 40 cm × 30 cm and designed from Plexiglass in a box shape [6]. After acclimatization, the mice were habituated with the apparatus for 2–3 min one day before the test. Initially, in the sample phase, two identical objects were placed in the two corners of the apparatus and the mice were allowed to freely explore the apparatus. The objects in the apparatus were explored by each animal either by touching the objects or keeping their noses within 2 cm of them. Test phase was started after 24 h of the sample phase in which one of the familiar objects was replaced by a new (novel) object. Mice were allowed again to freely explore both the objects in a similar way as in sample phase, and the time spent by each animal exploring the novel and familiar object (F) was recorded.

The following formula was used for calculation of discrimination index.

$$\mathrm{D1}=\frac{N—F\;}{N+F}$$

D1 = discrimination index; N = novel object; F = familiar object

### 4.6. Assessment of Biochemical Parameters and Biomarker Study

After the behavioral study, the mice were sacrificed by decapitation, providing a quick and painless death by cervical dislocation. The isolated brain of each animal in chilled phosphate buffer saline was subjected into biomarker assays using AChE and BuChE enzymes [50].

#### 4.6.1. Cholinesterase Activity

Mice were sacrificed within 24 h of the behavior study; brains were dissected and stored at −80 °C. The bilateral hippocampus was homogenized before the assay into volumes of 0.01 M phosphate buffer (2.7 mM KCl, 137 mM NaCl, 2 mM KH2PO4, 10 mM Na2HPO4) and centrifuged at 4 °C for 30 min at 12,000× *g*. Supernatant was used for the measurement of cholinesterase (AChE and BuChE) activity.

##### Acetylcholinesterase (AChE) Activity

Acetylcholine/Acetyl cholinesterase assay Kit was used for the determination of AChE activity, according to the standard procedure. The assay was carried out using 96-well plate, and every reaction comprised of 100 µL sample, 200 µM Amplex Red reagent, 50 µM of acetylcholine, 1 U/mL horseradish peroxidase (HRP) and 0.1 U/mL choline oxidase in buffer solution. Incubation period for the reaction mixture was 20 min at room temperature in the dark. The fluorescence intensity at 560 nm excitation and 580 nm emission wavelength was measured [12].

##### Butyrylcholinesterase (BuChE) Activity

Butyrylcholinesterase (BuChE) activity of the synthesized compounds were measured according to Ellman method with slight modification [51]. BuSCh was hydrolyzed by BuChE and choline iodide produced, which reacted with DNTB, and then the production of TNB. The TNB was quantitated by calorimetry that indicated the BuChE activity. BuChE assay was accomplished in 96-well plate and every reaction mixture contained 40 µL sample, 80 µL DTNB (0.25 mg/mL) and 70 µL BuSCh (7.5 mM). The absorbance was determined at 37 °C after incubation for 60 min.

### 4.7. Statistical Analysis

The measured data was presented in mean ± SEM and analyzed statistically using One-way ANOVA by applying Dunnet’s multiple comparison tests on Graph Pad Prism Software 5.01.

## 5. Conclusions

The current study concluded that curcumin-based compounds with various functionalities can produce memory-enhancing effects. The six curcumin analogs (**1a–1f**) were synthesized, characterized and tested in vitro for cholinesterase inhibitory effects along with preliminary in vivo behavioral investigation. These compounds were further evaluated for cholinesterase inhibitory potential in the ex vivo analysis. The synthesized curcumin analogs demonstrated significant in vitro cholinesterase inhibitory activity, promising in vivo and ex vivo memory-enhancing effects, which was validated by molecular docking studies. These analogs improved memory in mice brains and restored the acetylcholine level by significantly inhibiting the activity of both AChE and BuChE enzymes. However, it needs further study to explore its exact mechanism.

## Figures and Tables

**Table 1:** In vitro cholinesterase inhibition potential of compounds (**1a**–**1f**).

Compound	AChEI (IC_50_ (nM))	BuChEI (IC_50_ (nM))
**1a**	2615.42	5347.16
**1b**	733.84	2159.08
**1c**	467.18	1356.14
**1d**	112.52	378.43
**1e**	3267.95	6635.82
**1f**	5839.96	9664.71
Donepezil	9.31	33.65

Donepezil was used as positive control. Data were expressed in mean ± SEM, (*n* = 3), and significantly different values were compared to positive control.

**Table 2:** The docking scores and binding energies of compounds (**1a–1f**) of the calculated IC_50_ values against AChE and BuChE.

Comp	AChEI (IC_50_ nM)	Docking Score	Binding Energy (GBVI/WSA)	BuChEI (IC_50_ nM)	Docking Score	Binding Energy (GBVI/WSA)
**1a**	2615.42	−7.371	−13.101	5347.16	−7.871	−13.207
**1b**	733.84	−8.236	−14.082	2159.08	−8.054	−14.749
**1c**	467.18	−10.427	−18.127	1356.14	−8.556	−15.217
**1d**	112.52	−12.089	−21.159	378.43	−10.962	−19.081
**1e**	3267.95	−7.391	−13.330	6635.82	−6.918	−12.652
**1f**	5839.96	−6.998	−12.146	9664.71	−6.202	−11.023

The lower scores showed more favorable pose in all the scoring functions. In the molecular docking studies, choosing between active and non-active molecules is based on binding energies and docking score. GBVI/WSA is a scoring function of estimated free energy from a given pose in ligand binding and represents Generalized-Born Volume Integral/Weighted Surface Area.

## Data Availability

All data contained within this article.

## Supplementary Materials

The following supporting information can be downloaded at: https://www.mdpi.com/article/10.3390/molecules27082468/s1, ^1^H NMR of synthesized curcumin analog **1a**, **1b**, **1d**, **1e** and **1f** and mass spectra of synthesized curcumin analog **1a**, **1b**, **1d** and **1e**.

## Sample Availability

Sample of the compounds are available. Will be provided on reasonable request.
