# Supplementary material for: Attenuation of Scopolamine-Induced Amnesia via Cholinergic Modulation in Mice by Synthetic Curcumin Analogs
Source: Molecules. 2022 Apr 11;27(8):2468. doi: 10.3390/molecules27082468 (PMC9029618; doi:10.3390/molecules27082468)

Proton NMR spectra of synthesized curcumin analog (**1a**)

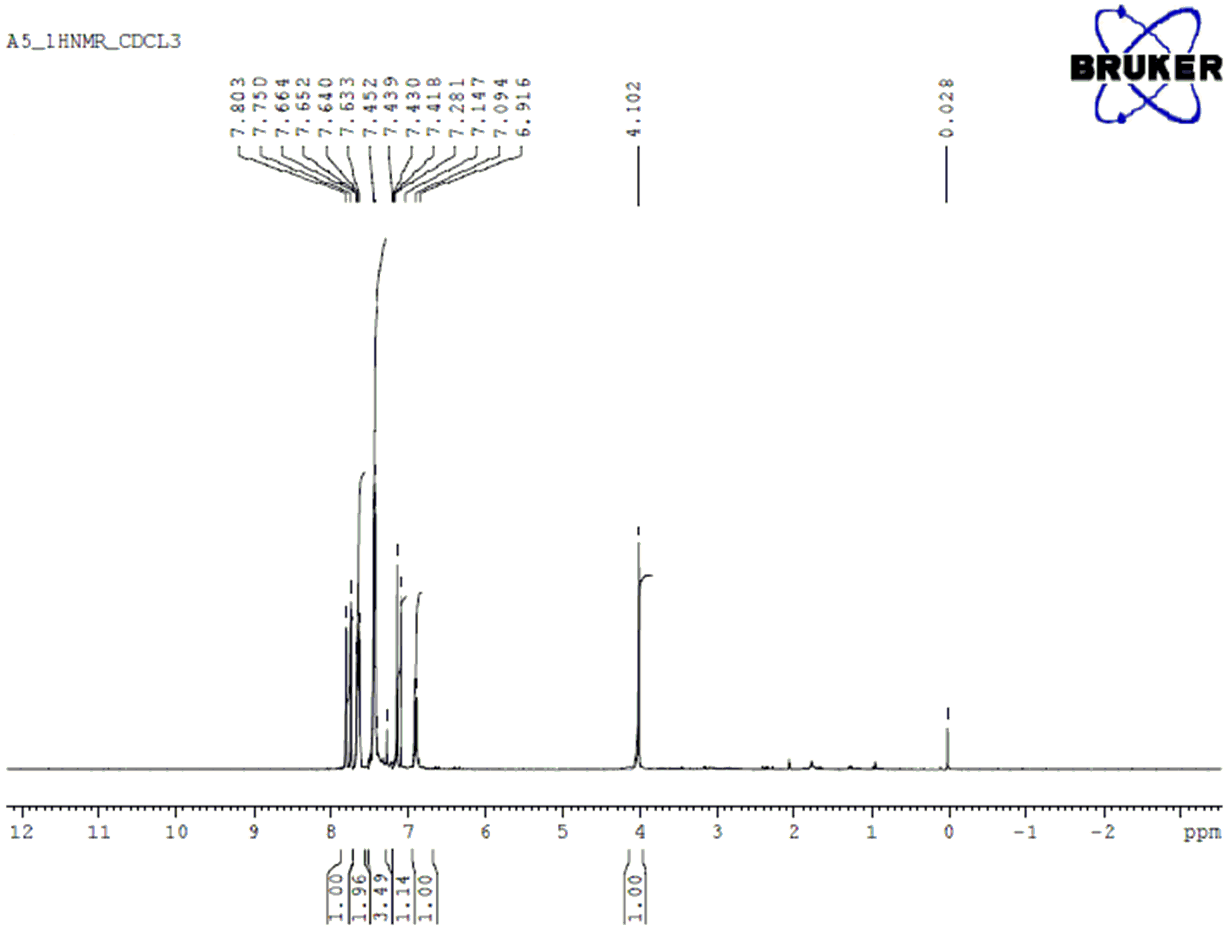

Proton NMR spectra of synthesized curcumin analog (**1b**)

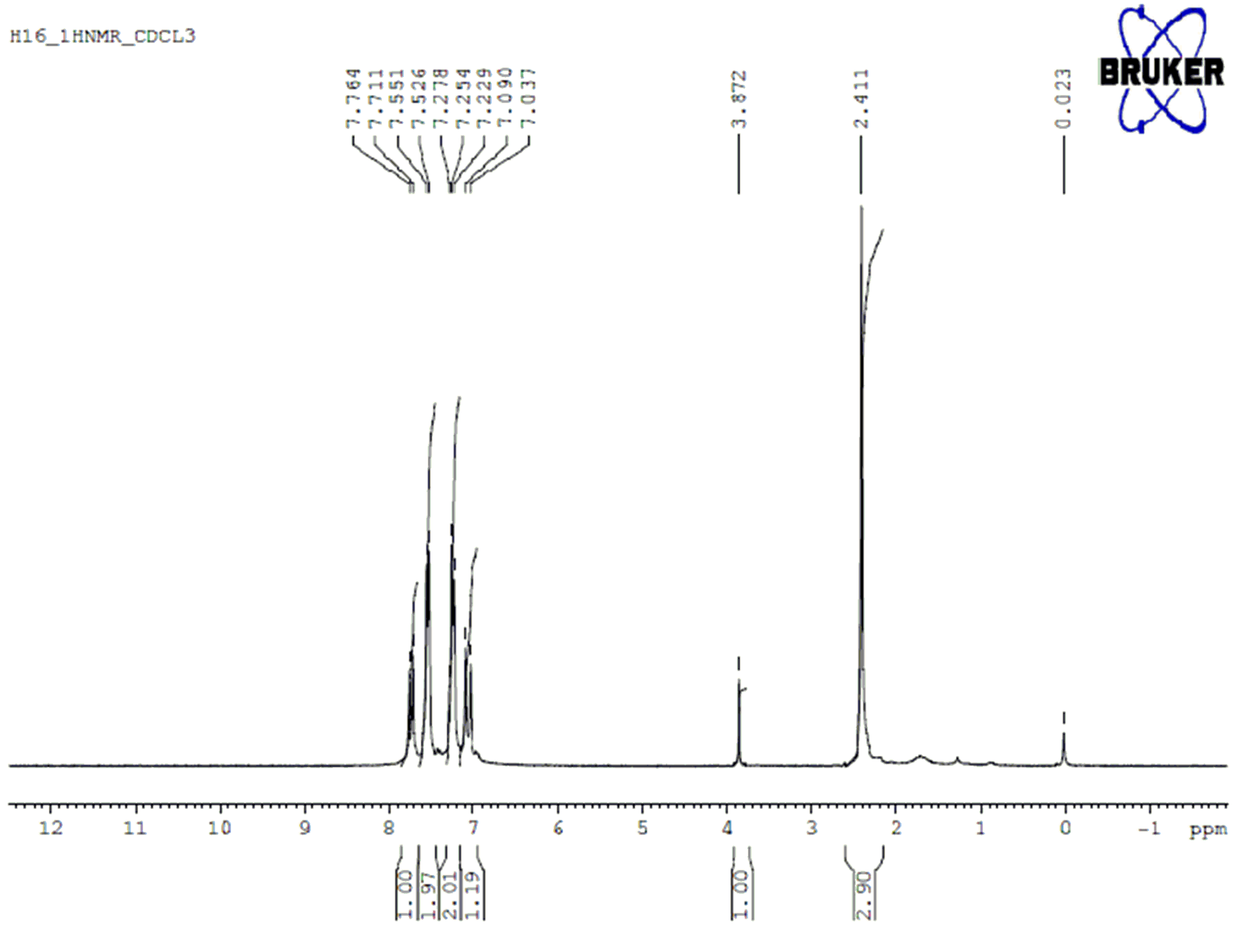

Proton NMR spectra of synthesized curcumin analog (**1d**)

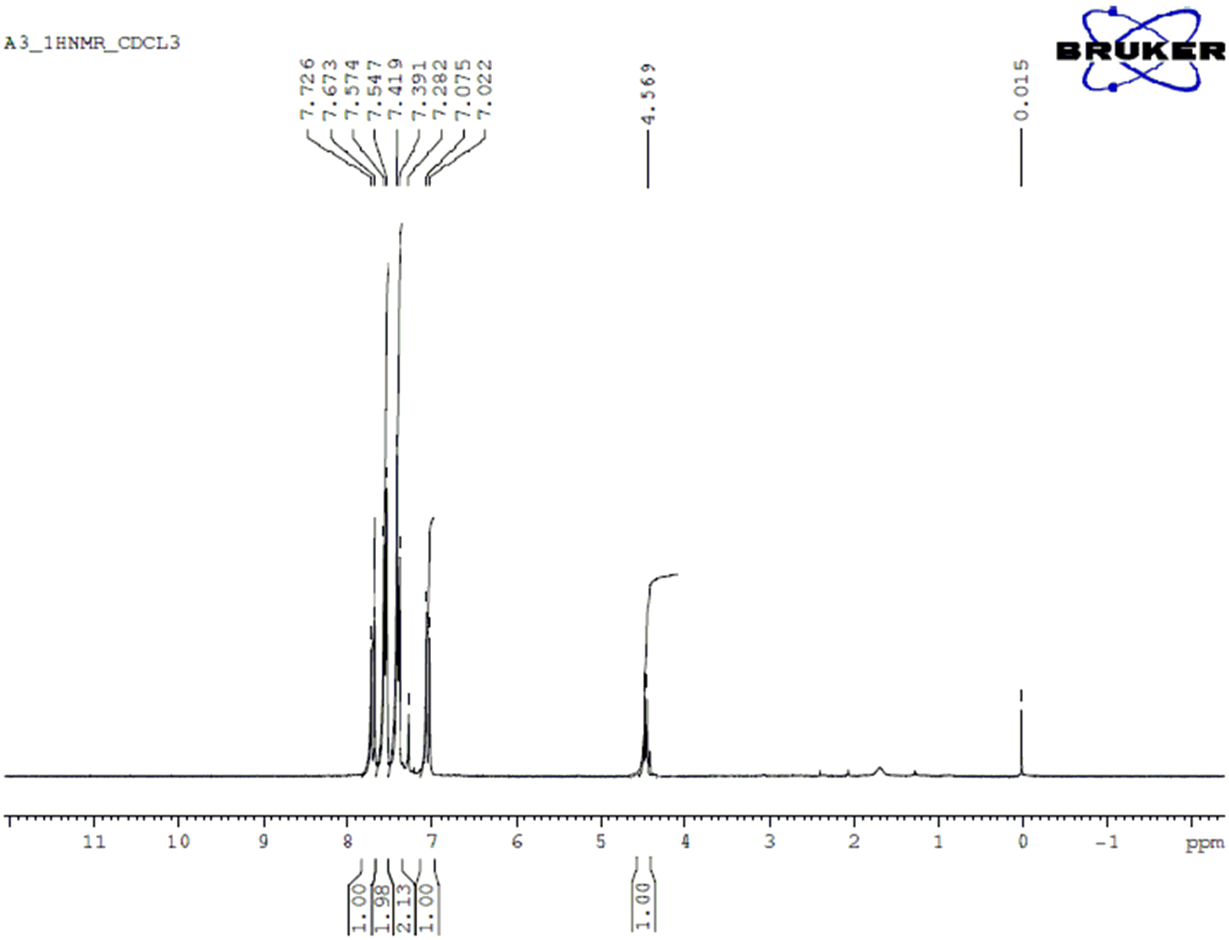

Proton NMR spectra of synthesized curcumin analog (**1e**)

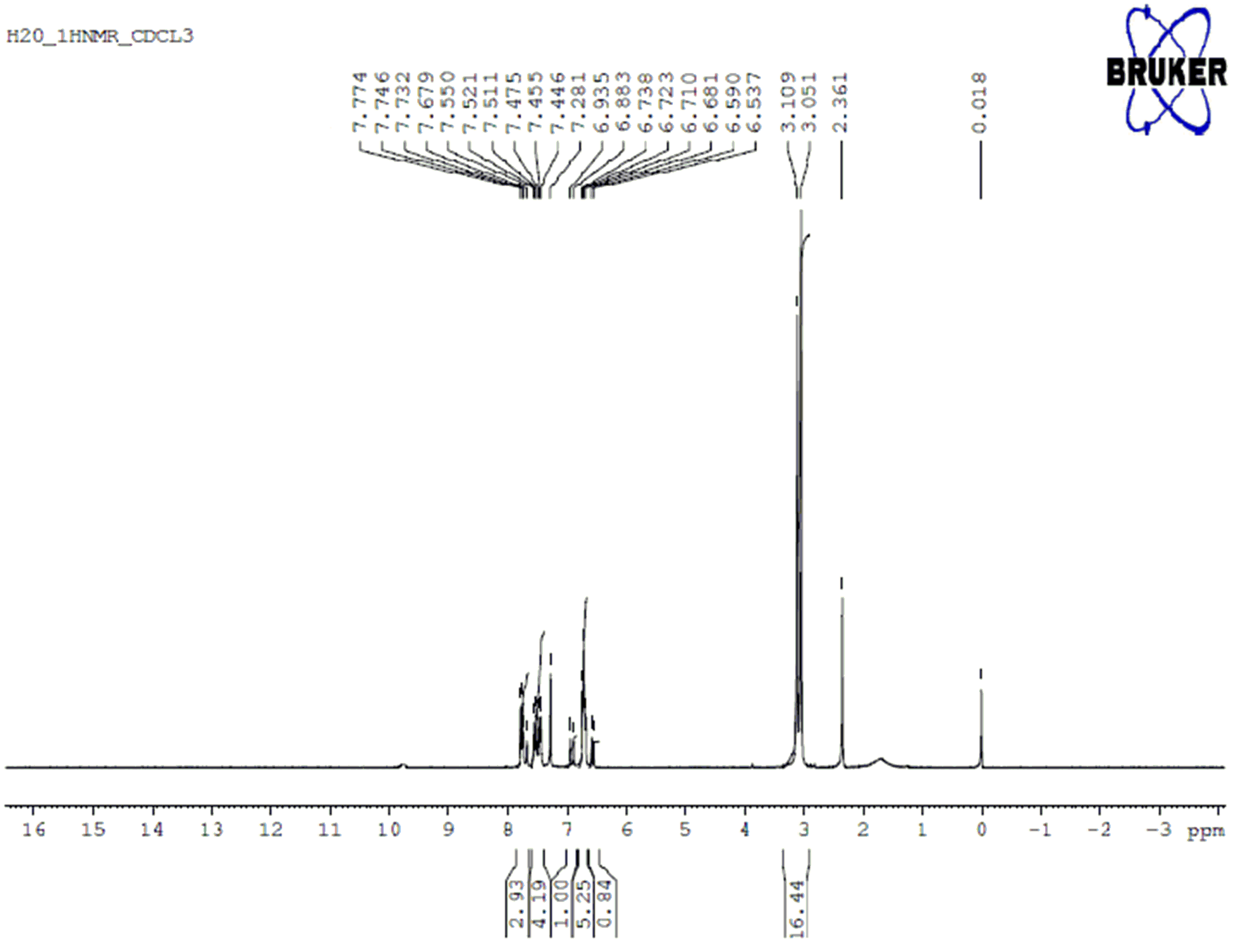

Proton NMR spectra of synthesized curcumin analog (**1f**)

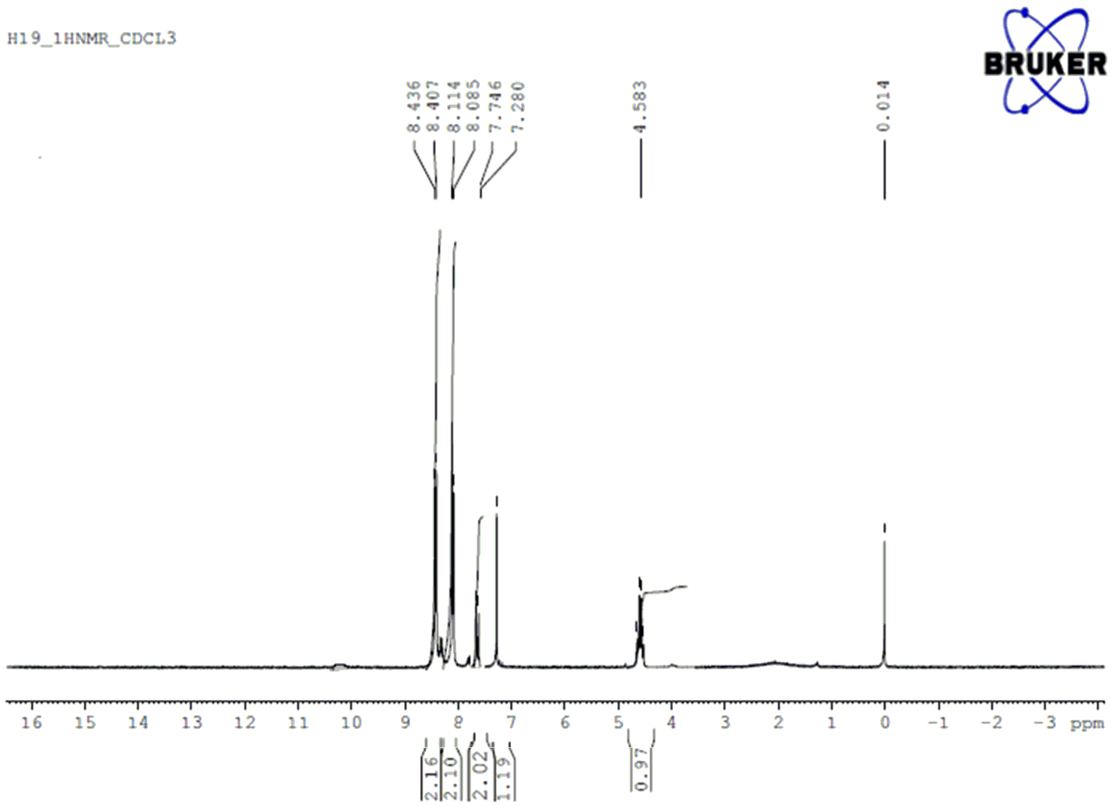

Mass Spectra

1a

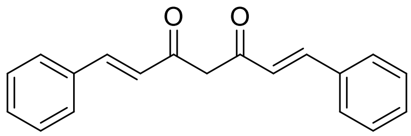

Exact Mass: 276.12

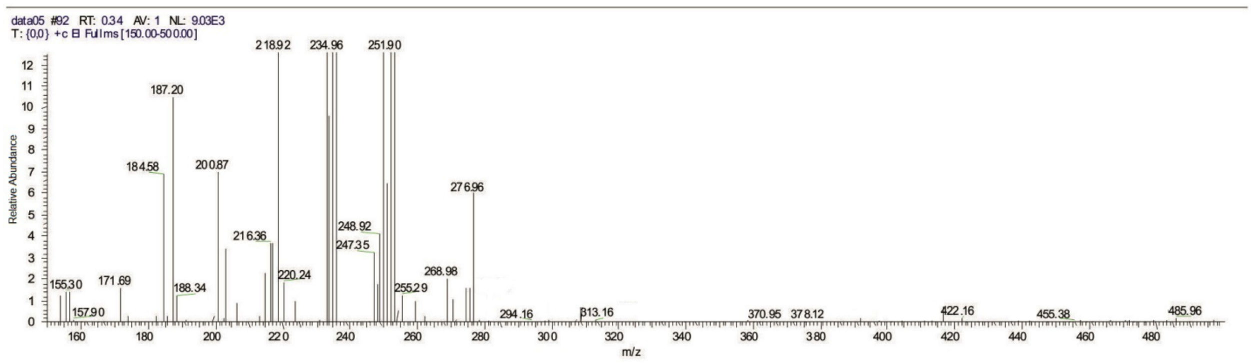

1b

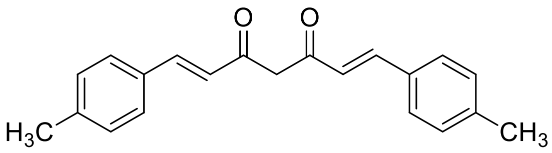

Exact Mass: 304.15

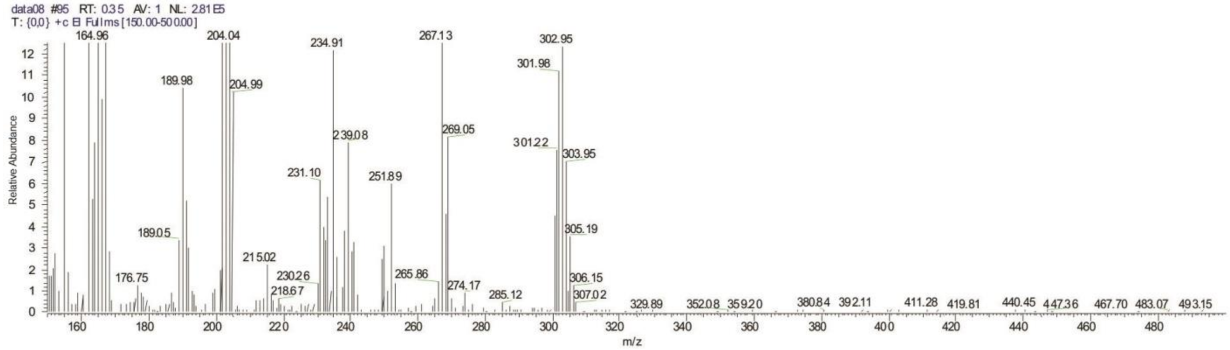

1d

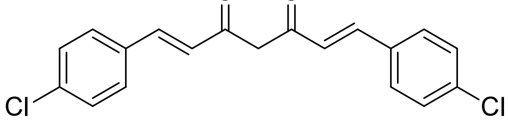

Exact Mass: 344.04

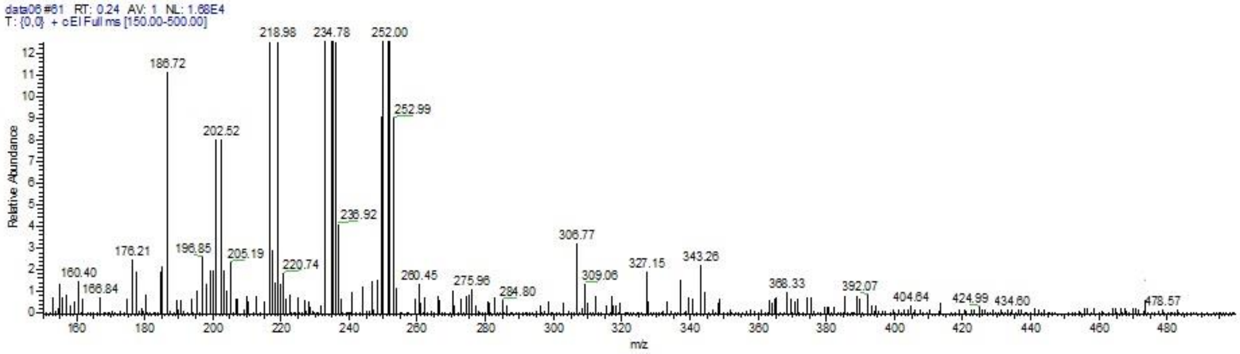

1e

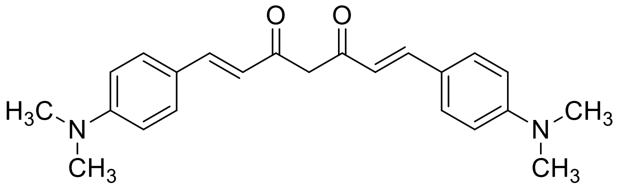

Exact Mass: 362.20

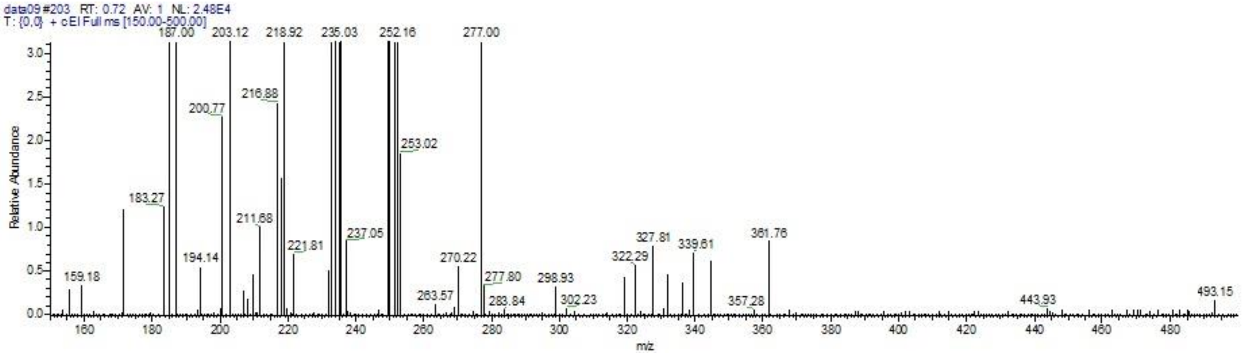

Supplement: Supplementary file 1 [file molecules-27-02468-s001.zip › molecules-1651564-supplementary.pdf]
